# Extrapulmonary features of bronchiectasis: muscle function, exercise capacity, fatigue, and health status

**DOI:** 10.1186/2049-6958-7-3

**Published:** 2012-06-11

**Authors:** Ozge Ozalp, Deniz Inal-Ince, Ebru Calik, Naciye Vardar-Yagli, Melda Saglam, Sema Savci, Hulya Arikan, Meral Bosnak-Guclu, Lutfi Coplu

**Affiliations:** 1 Faculty of Health Sciences, Department of Physiotherapy and Rehabilitation, Hacettepe University, 06100, Samanpazari, Ankara, Turkey; 2 Dokuz Eylul University, School of Physiotherapy and Rehabilitation, 35340, Inciralti, Izmir, Turkey; 3 Gazi University, Faculty of Health Sciences, Department of Physiotherapy and Rehabilitation, Emniyet Mah., Muammer Yasar Bostanci Cad. No: 16, 06500, Besevler, Ankara, Turkey; 4 Faculty of Medicine, Department of Chest Medicine, Hacettepe University, 06100, Sihhiye, Ankara, Turkey

**Keywords:** Bronchiectasis, Exercise, Muscle function, Health status

## Abstract

**Background:**

There are limited number of studies investigating extrapulmonary manifestations of bronchiectasis. The purpose of this study was to compare peripheral muscle function, exercise capacity, fatigue, and health status between patients with bronchiectasis and healthy subjects in order to provide documented differences in these characteristics for individuals with and without bronchiectasis.

**Methods:**

Twenty patients with bronchiectasis (43.5 ± 14.1 years) and 20 healthy subjects (43.0 ± 10.9 years) participated in the study. Pulmonary function, respiratory muscle strength (maximal expiratory pressure – MIP - and maximal expiratory pressure - MEP), and dyspnea perception using the Modified Medical Research Council Dyspnea Scale (MMRC) were determined. A six-minute walk test (6MWT) was performed. Quadriceps muscle, shoulder abductor, and hand grip strength (QMS, SAS, and HGS, respectively) using a hand held dynamometer and peripheral muscle endurance by a squat test were measured. Fatigue perception and health status were determined using the Fatigue Severity Scale (FSS) and the Leicester Cough Questionnaire (LCQ), respectively.

**Results:**

Number of squats, 6MWT distance, and LCQ scores as well as lung function testing values and respiratory muscle strength were significantly lower and MMRC and FSS scores were significantly higher in patients with bronchiectasis than those of healthy subjects (*p* < 0.05). In bronchiectasis patients, QMS was significantly associated with HGS, MIP and MEP (*p* < 0.05). The 6MWT distance was significantly correlated to LCQ psychological score (*p* < 0.05). The FSS score was significantly associated with LCQ physical and total and MMRC scores (*p* < 0.05). The LCQ psychological score was significantly associated with MEP and 6MWT distance (*p* < 0.05).

**Conclusions:**

Peripheral muscle endurance, exercise capacity, fatigue and health status were adversely affected by the presence of bronchiectasis. Fatigue was associated with dyspnea and health status. Respiratory muscle strength was related to peripheral muscle strength and health status, but not to fatigue, peripheral muscle endurance or exercise capacity. These findings may provide insight for outcome measures for pulmonary rehabilitation programs for patients with bronchiectasis.

## Background

Bronchiectasis is a chronic pulmonary disease which is caused by the destruction of elastic tissue and smooth muscles on bronchial walls due to repeated severe infection or inflammation and characterized by abnormal permanent dilatation [1]. Previous studies determined that skeletal muscle (respiratory and limb) abnormalities in addition to respiratory tract involvement are present in chronic inflammatory pulmonary diseases [2]. In these diseases, muscle dysfunction is caused by inflammation, gas exchange abnormalities, electrolyte imbalance, inactivity, malnutrition and drugs [2]. Peripheral muscle weakness and lack of endurance negatively affect the exercise capacity and perception of fatigue in chronic respiratory airway diseases [2-4].

Bronchiectasis is a common health problem for adult populations in developing countries [5]; however there is a limited number of studies investigating exercise capacity in adult patients [6-9]. Peripheral muscle function has not been studied in adult patients with bronchiectais. No study was found by the authors in the literature that provided findings in comparison with the results of healthy individuals. In the limited number of studies found, physical dimension of quality of life and localization of the disease were reported to affect exercise capacity [8], depression was related to exercise performance [7], and perception of dyspnea was related to exercise capacity [6]. No study associating peripheral and respiratory muscle weakness in bronchiectasis with exercise capacity, respiratory function and health status was found in the literature. Therefore, the aim of the present study was to evaluate extrapulmonary manifestations of bronchiectasis namely peripheral muscle function, exercise capacity, fatigue and health status of adult patients with bronchiectasis in comparison with healthy individuals to provide data about deviation from the normal values of these characteristics in individuals with bronchiectasis.

## Methods

The study included 20 adult patients with bronchiectasis diagnosed in the Department of Chest Medicine by high resolution computed tomography (10 F, 10 M) and 20 healthy subjects (13 M, 7 F). Consecutive patients who were referred to the Cardiopulmonary Rehabilitation Unit participated in the study. The mean (± SD) number of lobes showing changes of bronchiectasis was 2.9 ± 1.0 (median: 3, range 1–5 lobes). The mean (± SD) time from the diagnosis was 12.8 ± 11.8 years (median: 8, range 1–33 years). Individuals who were using antibiotics, had neurologic or orthopedic disease, advanced heart failure, or an acute exacerbation within the last three weeks were not included in the study. The control group was composed of individuals who had no known systemic, orthopedic or neurologic disease, aged between 18–64 years, could walk and cooperate, and were voluntary subjects among the relatives of researchers and academic personnel. Participants were informed about the aim and scope of the study, and written consent was obtained from each participant. This prospective study was approved by Ethical Committee of Hacettepe University.

Physical, physiological and socio-demographic characteristics of the subjects were recorded. Body Mass Index (BMI) was calculated by the formula of weight/height^2^ (kg/m^2^). Fat free mass (FFM) was determined using the skinfold method (Skinfold Caliper, Holtain Ltd, Crosswell, UK) from biceps, triceps, subscapular, and suprailiac regions. Measurements were repeated three times and the mean of three measurements was used [10].

The pulmonary function test was performed in sitting position using a spirometer (Spirolab III Medical International Research, Rome, Italy). Forced vital capacity (FVC), forced expiratory volume in one second (FEV_1_), FEV_1_/FVC, peak expiratory flow (PEF) and forced expiratory flow 25–75% (FEF_25–75%_) values were recorded. Parameters of the respiratory function test were expressed as percentages of the expected values in accordance with the subject’s age, height, body weight and sex [11].

Respiratory muscle strength was measured with a portable electronic mouth pressure device (Micro Medical MicroMPM, UK). Maximal inspiratory and expiratory mouth pressures (MIP and MEP) were recorded. MIP was measured at residual volume, while MEP was determined at total lung capacity. Measurements were repeated three times to avoid any difference larger than 10% or 10 cm H_2_O and the best measurement analysis was chosen. Values were expressed as percentages of the expected values in terms of age and sex [12].

Perception of dyspnea was evaluated by the Modified Medical Research Council (MMRC) dyspnea scale. The MMRC is a 0–4 point category scale which selects the best expression to define the dyspnea levels among five expressions related to dyspnea [13].

Peripheral muscle strength of the participants (quadriceps, shoulder abductor and hand grip strength) was evaluated by digital dynamometer (JTECH, Medical Commander Powertrack II, USA). Measurements were repeated three times for the dominant side [14]. The highest value of the measurements was expressed in Newton (N). Peripheral muscle endurance was assessed by a squat test. In the squat test, subjects were asked to move from a standing position to a squatting position. The number of squats completed in thirty seconds was recorded for analysis [14].

For the six-minute walk test (6MWT), subjects were asked to walk along a flat corridor at their walking speed as quickly as possible for six minutes [15]. In pre- and post-test periods, values of oxygen saturation were measured by pulse oximeter (KPTS-01, Seoul, Korea). Heart rate, blood pressure and respiratory rate were also recorded. Perception of fatigue and dyspnea in pre- and post-test periods was assessed with the Modified Borg Scale, which is a 0–10 point category scale [16]. Upon completion of the test, the distance covered in the 6-minute walk was recorded in meters. The 6MWT was applied twice with an interval of half an hour on the same day. For each patient, the longer walking distance of two tests was used for statistical analysis [16]. The 6MWT distances expressed as percentages of expected values from age and sex (6MWT% of distance) were calculated [17].

The Fatigue Severity Scale (FSS) was used to estimate the fatigue level of the subjects [18]. The FSS is a one-dimensional scale developed to assess fatigue and composed of nine items. The subject is asked to score each expression between 1 (completely agree) and 7 (completely disagree). In the FSS, ≥ 4 points implies the presence of severe fatigue [18].

Health status was assessed using the Leicester Cough Questionnaire (LCQ). The LCQ is composed of 19 items and includes physical (8 items), psychological (7 items) and social (4 items) sub-dimensions. Each item is scored between 1 (always) and 7 (never). The score of each sub-dimension ranges between 1 and 7. Total score ranges between 3 and 21. Low scores on the LCQ indicate a higher effect of coughing on the subject [19].

SPSS 15.0 packet software was used for the statistical analysis [20]. Variables were expressed as mean ± standard deviation, frequency and percentages. The Shapiro Wilk test was used to analyze the suitability of variables to normal distribution [21]. The Student’s *t*-test was used for the comparison of variables suitable for normal distribution. Mann–Whitney *u*-test was used to compare nonparametric variables not suitable for normal distribution. Comparison of the variables determined by counting was performed by the Chi-Square test. Corrected correlations (for age, weight, height and sex) were used to investigate association among the variables. The level of significance was set to *p* < 0.05.

## Results

Physical and demographic characteristics of patients with bronchiectasis and healthy subjects were similar in the study (*p* > 0.05, Table 1). FVC, FEV_1_, FEV_1_/FVC, PEF, FEF_25-75%_, MIP, and MEP were significantly lower in patients with bronchiectasis compared to healthy subjects (*p* < 0.05, Table 1). The MIP of four subjects (20%) and MEP value of 15 subjects (75%) were lower than 80% of their predicted value. The perception of dyspnea in subjects with bronchiectasis determined using the MMRC was significantly higher than that in healthy subjects (*p* < 0.05, Table 1).

**Table 1:** Characteristics of patients with bronchiectasis and healthy subjects

Characteristics	Bronchiectasis (n = 20)	Healthy (n = 20)	p
Age (years)	43.5 ± 14.1	43.0 ± 10.9	0.891
Sex (male/female)	10/10	13/7	0.52
Height (cm)	165.6 ± 8.7	164.7 ± 7.6	0.72
Body weight (kg)	68.8 ± 16.4	75.4 ± 12.9	0.17
Body mass index (kg/m^2^)	24.8 ± 4.6	27.96 ± 5.2	0.06
FFM (kg)	50.4 ± 11.4	56.23 ± 10.4	0.096
FVC (%)	70.4 ± 15.9	100.3 ± 12.7	< 0.0001*
FEV_1_ (%)	62.5 ± 20.0	97.3 ± 10.7	< 0.0001*
FEV_1_/FVC (%)	73.4 ± 14.8	80.9 ± 5.4	0.045*
PEF (%)	63.9 ± 23.3	101.8 ± 15.2	< 0.0001*
FEF_25–75%_ (%)	45.4 ± 24.0	80.2 ± 17.2	< 0.0001*
MIP (cmH_2_O)	97.5 ± 30.3	115.8 ± 19.9	0.030*
%MIP	99.0 ± 28.1	109.4 ± 21.4	0.019*
MEP (cmH_2_O)	125.8 ± 33.6	170.0 ± 47.0	0.003*
%MEP	68.0 ± 13.8	85.7 ± 23.2	0.006*
MMRC (0–4)	1.55 ± 0.60	0.20 ± 0.52	< 0.0001*

*p < 0.05.

Table 1 legend - FEF_25-75%_, forced expiratory flow 25–75%; FEV_1_, forced expiratory volume in one second; FFM, fat free mass; FVC, forced vital capacity; MIP, maximal inspiratory pressure; MEP, maximal expiratory pressure; MMRC, Modified Medical Research Council dyspnea scale, PEF, peak expiratory flow rate.

Although no significant difference was found between subjects with bronchiectasis and healthy subjects regarding peripheral muscle strength measures (*p* > 0.05, Table 2), quadriceps muscle strength tended to be lower in the group with bronchiectasis (*p* = 0.050, Table 2). Quadriceps muscle strength was significantly associated with hand grip strength (r = 0.52, *p* = 0.037), MIP (r = 0.58, *p* = 0.020) and MEP (r = 0.66, *p* = 0.029) in patients with bronchiectasis. The SAS was significantly correlated with MEP (r = 0.53, *p* = 0.035), and HGS was significantly associated with MIP (r = 0.52, *p* = 0.040).

**Table 2:** Peripheral muscle function, exercise capacity, fatigue and health status in patients with bronchiectasis and healthy subjects

	Bronchiectasis (n = 20)	Healthy (n = 20)	p
Peripheral muscle strength			
Knee extension (N)	266.7 ± 63.3	310.4 ± 73.0	0.050
Shoulder abduction (N)	158.6 ± 56.7	183.0 ± 50.6	0.15
Hand grip (N)	176.9 ± 62.0	198.7 ± 54.5	0.18
Peripheral muscle endurance			
Squats (n)	15.80 ± 3.28	22.50 ± 4.43	< 0.0001*
Fatigue			
Fatigue Severity Scale	4.66 ± 1.67	3.37 ± 1.53	0.007*
Exercise capacity			
6MWT distance (m)	559.2 ± 98.7	636.0 ± 74.3	0.008*
6MWT%	90.5 ± 14.3	105.6 ± 12.5	0.001*
Borg-dyspnea	2.20 ± 1.90	0.07 ± 0.24	< 0.001*
Borg-fatigue	1.77 ± 1.89	0.92 ± 1.43	0.26
Borg-quadriceps fatigue	1.65 ± 1.89	0.95 ± 2.30	0.16
Health status			
LCQ total	14.67 ± 3.88	19.43 ± 1.33	< 0.0001*
LCQ physical	4.51 ± 1.37	6.53 ± 0.75	< 0.0001*
LCQ psychological	4.81 ± 1.12	6.01 ± 0.32	< 0.0001*
LCQ social	5.33 ± 1.65	6.87 ± 0.32	< 0.0001*

*p < 0.05.

Table 2 legend - 6MWT, six minute walk test; LCQ, Leicester Cough Questionnaire.

The number of squats was significantly lower in subjects with bronchiectasis compared to healthy subjects (*p* < 0.05, Table 2). It was not significantly related with any of the variables measured (*p* > 0.05).

The 6MWT distance and 6MWT% distance were significantly lower in subjects with bronchiectasis when compared to the healthy group, while exercise dyspnea perception was significantly higher in subjects with bronchiectasis (*p* < 0.05, Table 2). No significant difference was noted between the two groups in maximal heart rate percentage, oxygen saturation, blood pressure, general fatigue or quadriceps fatigue perceptions recorded during the test (*p* > 0.05). The 6MWT distance was significantly related to LCQ psychological score (r = 0.52, *p* = 0.042) in patients with bronchiectasis.

Thirteen patients (65%) with bronchiectasis reported having severe fatigue. The FSS value was significantly higher in the bronchiectasis compared to the healthy group (*p* < 0.05, Table 2). It was significantly associated with LCQ physical score (r = −0.56, *p* = 0.024), LCQ total score (r = −0.50, *p* = 0.047) and MMRC score (r = 0.53, *p* = 0.043).

The LCQ total score and the scores of physical, psychological and social sub-dimensions were significantly lower in the bronchiectasis group than in healthy subjects (*p* < 0.0001, Table 2). The LCQ total score was significantly related to FSS (r = −0.50, *p* = 0.047), and physical score was significantly and inversely related to FSS (r = −0.56, *p* = 0.024). The LCQ psychological score was significantly associated with MEP (r = 0.51, *p* = 0.044) and 6MWT distance (r = 0.52, *p* = 0.042).

## Discussion

The present study demonstrated that bronchiectasis affects peripheral muscle endurance, exercise capacity, fatigue, and health status in addition to its effects on pulmonary function, inspiratory and expiratory muscle strength, and dyspnea perception in adult subjects with bronchiectasis. Exercise capacity is related to health status. Fatigue is affected by dyspnea perception and health status.

Different levels of respiratory dysfunction could be seen in bronchiectasis [5,22]. Mucociliary clearance dysfunction, bronchial inflammation and infection, irreversible bronchial dilatation, and destruction in elastic and muscular components of bronchial walls decreased expiratory air flow in the lungs [1,5,23]. Effects on pulmonary function could be seen as obstructive, restrictive or of a mixed type [5,22]. According to the results of pulmonary function tests in the study, mild airflow obstruction was determined in large airways, while severe obstruction was present in medium or small airways. These findings indicate that pulmonary function in our patients with bronchiectasis showed a pattern of airflow limitation probably due to chronic inflammation and destroyed bronchial wall [5,22-24].

In the present study, MIP and MEP were decreased in bronchiectasis. The MIP was lower than 80% of the expected value in four subjects, and MEP was lower in 15 subjects. This finding is considered significant as it demonstrates the presence of respiratory muscle weakness which is especially evident for expiratory muscles in subjects with bronchiectasis. Respiratory muscle strength has been measured for adult patients with bronchiectasis in a limited number of studies in literature [9,25,26]. In the present study, the obtained MIP and MEP values were higher than the measurements reported by Moran et al. (MIP: 74.20 cmH_2_O; MEP: 104.30 cmH_2_O), Newall et al. (MIP: 73.86 cmH_2_O; MEP: 86.83 cmH_2_O), and Murray et al. (MIP: 43.5 cmH_2_O; MEP: 68.5 cmH_2_O) [9,25,26] probably due to the inclusion of younger patients with bronchiectasis who had better respiratory function.

Dyspnea is seen in 60% of patients with bronchiectasis [5,24]. We used MMRC to evaluate dyspnea, which is considered one of the major factors defining bronchiectasis [27] and affects the survival along with airway obstruction, pulmonary hyperinflation and frequency of disease [1]. In the present study, dyspnea perception was evidently higher in patients with bronchiectasis when compared to the healthy group, which demonstrates that MMRC could distinguish the dyspnea level between subjects with bronchiectasis and healthy groups.

In a comprehensive evaluation of skeletal muscle function, peripheral muscle strength and peripheral muscle endurance must be considered together [3]. In this regard, the present study is the first in the literature to investigate peripheral muscle strength and endurance in adult patients with bronchiectasis. In our study, isotonic muscle strength of quadriceps tended to be lower than the values of the healthy group, while the peripheral muscle endurance was evidently reduced. Quadriceps muscle strength was found to be related to inspiratory and expiratory muscle strength and peripheral muscle endurance in patients with bronchiectasis. Peripheral muscle endurance was associated with expiratory muscle strength, upper and lower extremity muscle strength, exercise capacity, and fatigue perception. However, MEP was the only variable having an independent relationship with quadriceps muscle strength, and quadriceps strength was the only variable having an independent association with peripheral muscle endurance. Skeletal muscles play an important role both in motor (ventilation, ambulation and postural control) and non motor functions (thermoregulation and systemic metabolism) [28]. These neural networks and the vulnerability of skeletal muscles - respiratory or peripheral - to systemic disorders [28] including chronic lung diseases may be responsible for the above mentioned associations we found in terms of muscle dysfunction in our bronchiectatic patients. Further study is needed to clarify the cause of this deteriorating peripheral muscle function.

In previous studies investigating exercise capacity of adult patients with bronchiectasis, exercise performance was reported to be reduced [6-9]. In the present study, exercise capacity was examined using 6MWT and it was found to be lower when compared to healthy subjects. The 6MWT distance was significantly related to expiratory muscle strength, peripheral muscle strength and endurance. Hand grip strength was the only variable having an independent association with 6MWT distance. Peripheral muscle strength is a strong determinant of 6MWT in chronic lung disease [29], and hand grip strength is thought to be representative of general strength [30]. The lower exercise capacity of subjects with bronchiectasis could be caused by increased mucus production, deterioration in respiratory function, decrease in respiratory muscle strength, impaired health related quality of life [8], and loss of peripheral muscle endurance.

In recent years, fatigue has been reported to be commonly present in chronic pulmonary disease. At clinical investigation, it was determined to be present in as many as 74% of patients [5,31]. In a study evaluating fatigue perception of subjects with bronchiectasis using a standardized questionnaire, fatigue sense was determined to be higher in patients with bronchiectasis who had higher anxiety and depression levels [7]. In the present study, severity of fatigue as estimated by the FSS (a standardized measurement method) was found to be increased in subjects with bronchiectasis compared to a healthy group, and it was concluded as being severe (FSS ≥ 4 points) in the majority of the patients. These findings prove that fatigue is an important symptom for patients with bronchiectasis similarly to patients with COPD. In our patients with bronchiectasis fatigue was associated with dyspnea and health status. This finding suggests that fatigue should be evaluated using standardized specific questionnaires since it may not be predicted from the clinical measures including both pulmonary and peripheral variables.

In the limited number of studies examining health status of patients with bronchiectasis, impaired health status was found using a disease-specific standard questionnaire [8,25,32-36]. Dyspnea perception, FEV_1_ and daily amount of produced mucus are the factors independently affecting health status in patients with bronchiectasis [33]. In the present study, the effect of coughing symptoms frequently observed in bronchiectasis on health status was examined using LCQ. The difference with respect to healthy subjects was concluded to be higher than 1.3 points, which is a clinically significant level [37]. According to these results, the health status of subjects with bronchiectasis is evidently affected by the disease. In our study, an impaired physical component of disease-specific health status was associated with increased fatigue and dyspnea perception. In general, impaired health status was influenced only by fatigue perception. These findings reveal that frequent subjective symptoms have an effect on health status in clinically stable adult patients with bronchiectasis.

In this study, we used healthy controls for the comparison of extrapulmonary features of muscle function, exercise capacity, fatigue, and health status in patients with bronchiectasis. Although skeletal muscles involvement is frequent in COPD patients, we did not use a control group consisting of COPD patients with the same obstruction but preferred to use healthy subjects, since investigations that lack a healthy control group are not able to provide documented differences in the extrapulmonary characteristics between individuals with *vs*. without bronchiectasis. In our study, healthy subjects provided data concerning deviation from the normal values. A search of similar articles in the literature revealed that they did not include any controls [5-8,31-35]. Since the aim of the study was not to compare two different obstructive diseases, we wanted to provide findings showing how bronchiectasis patients’ extrapulmonary status was affected by their respiratory status.

## Conclusions

In conclusion, we found that perceptions of dyspnea and fatigue, peripheral muscle endurance, exercise capacity and health status are affected by the disease in subjects with bronchiectasis in addition to pulmonary function, inspiratory and expiratory respiration muscle strength. These extrapulmonary features of bronchiectasis may provide insight for outcome measures for pulmonary rehabilitation programs in patients with bronchiectasis.

## Competing interests

The authors declare that they have no competing interests.
